# Association Study between Functional Polymorphisms of *MMP9* Gene Promoter and Multiple Sclerosis Susceptibility in an Iranian Population

**Published:** 2019-09

**Authors:** Sima SABBAGH, Zakiye NADEALI, Leila DEHGHANI, Vahid SHAYGANNEJAD, Majid REZVANI, Masih SABOORI, Hamid GANJI, Soheil TAHANI, Farzaneh DEHGHANI, Saman HOSSEINZADEH, Elnaz MARZBANI, Peiman SHAFIEI

**Affiliations:** 1. Isfahan Neurosciences Research Center, Al-Zahra Research Institute, Isfahan University of Medical Sciences, Isfahan, Iran; 2. Medical Genetics Laboratory, Al-Zahra University Hospital, Isfahan University of Medical Sciences, Isfahan, Iran; 3. Department of Tissue Engineering and Regenerative Medicine, School of Advanced Technologies in Medicine, Shahid Beheshti University of Medical Sciences, Tehran, Iran; 4. Department of Neurosurgery, School of Medicine, Isfahan University of Medical Sciences, Isfahan, Iran

**Keywords:** Multiple sclerosis, *MMP-9* gene, Polymorphism, Association study

## Abstract

**Background:**

Matrix metalloproteinase-9 (*MMP-9*) polymorphisms, C-1562 T and -90 (CA) n repeats, which influence transcriptional activity of this gene, are proposed to play a role in MS susceptibility and its development. In the present study, the possible association of *MMP-9* polymorphisms in Iranian MS patients is studied.

**Methods:**

Association of *MMP-9* mentioned gene polymorphisms with MS susceptibility was evaluated in unrelated Iranian subjects referred to Al-Zahra Hospital, Isfahan, Iran during 2014 to 2017.

**Results:**

-1562 T allele of *MMP-9* was associated with increased MS risk. However, we found no overall significant effect of −90 (CA)n repeat on MS susceptibility.

**Conclusion:**

For as much as *MMP-9* molecule is a potential target for MS therapy, to determine whether any of *MMP-9* polymorphisms influence MS susceptibility in Iranian MS patients or not, concerning the significant influence of T allele on MS susceptibility and the non-significant association regarding CA repeats, further research is needed before proposing any definite conclusion.

## Introduction

Multiple sclerosis (MS) is a chronic disabling disease of the central nervous system (CNS) (1, 2). The episodic phase of relapsing-remitting (RR) MS is characterized by the breakdown of the blood-brain barrier (BBB), autoreactive inflammatory cells migrating into the CNS and demyelination (3, 4).

Matrix metalloproteinases (MMPs) are proteolytic enzymes implicated in many aspects of human development and tissue remodeling. These enzymes, particularly Matrix metalloproteinase-9 (*MMP-9*), are the major participants in breakdown of the blood-brain barrier (BBB) in MS (5, 6). *MMP-9*, also known as gelatinase B, degrades various components of the extracellular matrix (ECM) (7). MMP9 plays a role in the inflammatory cells influx in the CNS and have been revealed to split human myelin basic protein (MBP) (8–10). An elevation in levels of *MMP-9* has been detected in serum and cerebrospinal fluid (CSF) of MS patients (11, 12). Moreover, *MMP-9* has been considered to be a therapeutic response biomarker to IFN-beta, a drug employed commonly MS treatment (13). In addition, *MMP-9* level can be a helpful marker for the assessment of clinical type, disability, and severity of the disease (14). The influence of genetic polymorphisms of *MMP-9* gene on MS susceptibility is sustained by the association of *MMP-9* with MS pathogenesis. Two functional polymorphisms influence *MMP-9* gene transcriptional activity; C–1562 T (rs3918242) and microsatellite (CA)n 13–25 (rs3222264) identified in the promoter region (15–18). The region in which microsatellite (CA)n is located near the −90 positions can be used as a binding site by a specific DNA regulatory protein. It also facilitates the opening of the double strand of DNA and its transcription by enabling the DNA to switch to a Z structure. Additionally, in vitro studies the highest activity has been presented; the longest repeat alleles of microsatellite (17, 19, 20). The single nucleotide polymorphism C–1562 T prevents the binding of a nuclear repressor protein to this region of the *MMP-9* gene promoter, then attending to raised *MMP-9* expression, as reported in vitro studies (21).

Due to the differences in the allelic pattern of these polymorphisms in various populations, more studies are needed on these population to reach thorough conclusions. In addition, assessing the role of *MMP-9* polymorphisms classified by the various subtypes seems to be beneficial.

The aim of this study was to examine the possible association of two polymorphisms of the *MMP-9* gene in patients with relapsing-remitting MS in the Iranian population. Then the relationship between the *MMP-9* polymorphisms and MS susceptibility was evaluated.

## Materials and Methods

### Subjects

This study was designed as a case-control study and a total of 205 unrelated Iranian subjects entered in it from 2014 to 2017. The study included 100 patients with relapsing-remitting (RR) MS referred to Al-Zahra Hospital in Isfahan, central Iran. Overall, 105 control subjects randomly were taken from the same general community of central regions of Iran, with no family history of MS, nor any other autoimmune diseases. The control group was matched for age, ethnicity, and gender to the MS group. Subject characteristics are shown in Table 1.

**Table 1:** Characteristics of patients with MS and controls

***Characteristics***	***Control(n=105)***	***MS(n=100)***	***P value***
**Sex**			
Female	64(61%)	63(63%)	0.763
Male	41(39%)	37(37%)	
Age (yr)	46.52±8.90	42.89±10.48	0.008
Disease duration (yr)	10.71±7.13		
EDSS score	2.24±1.56		

Data are shown n (%) or Mean ± SD

Medical history, physical examination record and informed consent of each subject were obtained before proceeding to later stages of the study. All patients were taken into study from Department of Neurology of the Isfahan University of Medical Sciences and were auscultate by the same specialist neurologist in a functional MS center. Expanded Disability Status Scale (EDSS) was used to assess disease severity and duration.

This study has been approved by Ethics Committee of Isfahan University of Medical Sciences (approved No. 293178); all subjects filled consent form.

### Genotyping

Blood samples were taken from MS patients with and controls. DNA extraction from blood samples by salting out method was obtained. Genotyping of the C/T polymorphism at position -1562 of the *MMP-9* gene promoter (Gene bank accession number M68343) was carried using Tetra primer ARMS PCR. Primers for amplification of the C/T polymorphism were designed using Primer1 software.

PCR amplification in a reaction volume of 25 μl was performed, including 100 ng of template DNA, 15 pmol of each inner primer, 5pmol of each outer primer, 100 mM MgCl2, 100 μM dNTP, 2.5 μL 10× buffer and 2.5U Taq DNA polymerase. Polymerase chain reaction (PCR) procedure started with preincubation at 94 °C for 5 min, followed by 30 cycles of denaturation (94 °C for 30 sec), annealing (64 °C for 30 sec), extension (72 °C for 59 sec) and final extension was for 7 min at 72 °C. The PCR products were separated on 2% agarose gel electrophoresis and analyzed.

The numbers of the CA repeats in the *MMP-9* gene promoter was determined using previously described primers (19).

The PCR reactions for CA repeats were performed in 25 μl volume including 100 ng of template DNA, 100 mM MgCl2, 50μM dNTP mixture, 15pmol of each primer, 2.5 μL 10× PCR buffer and 2.5U Taq DNA polymerase. Amplification was carried out using initial denaturation at 94 °C for 5 min, followed by 35 cycles of denaturation (94 °C for 60 sec), annealing (69 °C for 60 sec), extension (72 °C for 60 sec) and final extension was for 7 min at 72 °C. The exact size of the alleles was determined by sequencing which uses an ABI 737 sequencer (Perkin Elmer/ABI, Life Technologies, Germany).

### Statistical Analysis

Finally, data were collected and all statistical analyses were performed using SPSS ver. 20.0 (SPSS Inc., Chicago, IL, USA).

Non-genetic risk factors between cases and controls were compared using t-test. The chi-square (χ^2^) test was used for independent segregation of the alleles of each genotype and for testing differences of the genotype frequencies in cases and controls. *P*-value <0.05 was considered to be statistically significant in each analysis. Odds ratios (OR) and 95% confidence intervals (CI) were calculated to assess the associations between individual genotypes of the C/T polymorphism and CA repeats and the risk of developing multiple sclerosis.

Indicators such as mean, standard deviation (SD), frequency and frequency percentage were used in descriptive analysis; in inferential statistics level, independent t-test, χ^2^ and logistic regression tests were applied.

## Results

The control group consisted of 105 healthy volunteers, 64 females (61%) and 41 males (39%), with a mean age of 46.52±8.90 yr (mean± S.D.). The study group consisted of 100 MS patients, 63 females and 37 males with a mean age of 42.89±10.48 yr. The mean duration of the disease was 10.71±7.13 yr and the mean EDSS of the disease was 2.24±1.56 yr (Table 1).

Genotype distribution and allele frequencies for 1562 C/T polymorphism in MS patients and controls are shown in Table 2. *MMP-9* promoter polymorphisms marker did not deviate from the Hardy–Weinberg equilibrium (HWE) in any group. A significant upper difference was observed in allele frequencies between MS patients (71.5%) compared to healthy volunteers (40%) in −1562C/T *MMP-9* gene polymorphism (*P*<0.001).

**Table 2:** Genotype and Allele distributions of the *MMP-9* promoter polymorphisms in MS and controls

***MMP-9 −1562 C/T***	***Control(n=105)***	***MS(n=100)***	***OR***	***CI 95%***	***P value***
Genotype	n(%)	n(%)			
TT	21(20)	54(54)	Reference		
CT	42(40)	35(35)	3.086	1.572–6.059	0.001
			3.923	1.888–8.151^a^	<0.001
CC	42(40)	11(11)	9.818	4.266–22.596	<0.001
			11.696	4.829–28.332 ^a^	<0.001
CT+CC	84(80)	46(46)	4.696	2.528–8.721	<0.001
			6.273	3.179–12.378	<0.001
Allele					
T	84(40)	143(71.5)	Reference		
C	126(60)	57(28.5)	3.763	2.490–5.687	<0.001

OR: odds ratio; CI=confidence interval.

a : Adjusted for age and sex

Additionally, frequency distribution of *MMP-9* genotypes has a significant relationship with MS disease; in such a way that CC+CT genotype opposed to TT showed a significant association with MS (OR (CI 95%): 4.696 (2.528–8.721), *P*<0.001). However, given to a significant difference of mean age between two study groups (*P*=0.008), confidence interval and odds ratio (ORs and CIs) were calculated for genotype and allele frequencies by logistic regression, while controlling for age and sex variables.

After adjusting such factors, significant association of genotype and allele of these polymorphisms with MS was preserved (*P*<0.001). However, given to a significant difference in the mean age of the study group, for genotype and allele frequencies, confidence interval and odds ratio (ORs and CIs) were calculated by logistic regression that confirm -1562C/T polymorphism were significantly associated with an increased risk of developing multiple sclerosis (*P*<0.001). Moreover, according to our findings, there were no significant associations between CA repeats and MS susceptibility (*P*>0.05) (Table 3).

**Table 3:** Genotype and Allele distributions of the *MMP-9* –90 (CA)_n_ promoter polymorphisms in MS and controls

***Microsatellite (CA)n***	***Control(n=105)***	***MS(n=100)***	***OR***	***CI 95%***	***P value***
**Genotype**	n(%)	n(%)			
L/L	39(37.1)	37(37)	Reference		
L/H	46(43.8)	41(41)	0.939	0.507–1.740	0.843
H/H	20(19)	22(22)	1.159	0.545–2.465	0.701
L/H+H/H	66(62.9)	63(63)	0.994	0.564–1.752	0.983
**Allele**					
L	124(59)	115(57.5)	Reference		
H	86(41)	85(42.5)	0.938	0.634–1.390	0.751

OR: odds ratio; CI=confidence interval.

CA repeat considered low: ≤20 and high: >20

## Discussion

This study is the first to elucidate the genetic impact of the *MMP-9–*1562 C/T and *–*90 (CA)n repeat polymorphisms on MS susceptibility in MS patients and controls in Iran. A nominally significant effect towards an increased -1562 T allele frequency in MS patients group was found. However, CA repeats did not demonstrate significant association with MS development. The matrix metalloproteinases (MMPs) represent a large family of extracellular matrix (ECM) degrading enzymes, among which *MMP-9* has shown a strong association with MS pathogenesis (15, 22). A raised activity in *MMP-9* has been associated with MS stages, disease activity and the severity of complications. In fact, elevated *MMP-9* levels correlate with the formation of new MS lesions (23). Different ethnic groups have a tendency to represent various patterns of *MMP-9* polymorphisms. As a result, following study plot, patient characteristics, and data analysis, the role of *MMP-9* polymorphism in susceptibility to MS could be varied in different populations (17, 19, 24, 25). Loss of binding of a nuclear repressor protein to the promoter of *MMP-9* gene is a result of *–*1562 C/T polymorphism in this region which leads to raised expression of *MMP-9*. In addition, this variant has been shown to affect promoter activity by in vitro studies, in which elevated promoter activity belongs to T allele (21). The comparison between findings in our sample with data of other studies shows that our results in the case of *–*1562 C/T polymorphism are similar to the results of the study on Italian populations (16). Contrary to our findings, some studies exhibited a decreased T allele frequency in MS patients in Serbian population (26–28). In addition, results of a recent meta-analysis manifested a significant association between *MMP-9* 1562 C/T polymorphism with other autoimmune diseases but it revealed no significant association between *MMP-9* 1562 C/T polymorphism and MS patients (29). Another polymorphism is the microsatellite (CA)n, consists of different repeat number of cytosine-adenine (13–27), which allele frequencies distribution culminates in two groups of CA≤14 and CA≥21–23. The expression of the gene to be controlled by different number of CA repeats. On the one hand, higher repeat alleles raise promoter activity and are significantly more frequent in MS patients’ population. On the other hand, comparing *MMP-9* promoter activity of these alleles with lower ones shows a 50% decline promoted by lower repeats (15, 19). Regarding the *–*90 (CA)n repeat polymorphism, the allele frequency with long microsatellite (i.e. 22 or more CA repeats), detected in our population from Isfahan City in Iran, was not significantly higher in patients than in control subjects.

We are conscious of the limitations of association studies. Given genetic variability among different populations and false positive and false negative findings, the contradiction of obtained results in different reports is prevalent. Specifically, false positive results might be caused by population stratification, misclassification and inappropriate statistical methods (16).

Some limitations of our study should be discussed as well. First, for comprehensive correlation analysis, this number of recruited patients was not great enough. As a matter of fact, in order to assess effects of *MMP-9* polymorphisms on MS accurately, larger sample size is needed to be recruited (19), but because of shortage in financial resources, we could not perform the test for more patients at the moment. Second, we select the method procedure due to our lack of facilities, so using more precise method would be beneficial.

Since 1562C/T gene polymorphism is reported diversely in different populations, and given to our results which are similar to an Italian study, 1562 T allele of *MMP-9* promoter could be used as a biomarker in Iranians particularly for people who are more vulnerable to MS, like those who have family history of this disease or live under special environmental conditions such as industrial workplaces. However, for CA repeats we did not reach an association in contrast to other studies; this does not allow us to report it as a biomarker for MS. Undoubtedly for the definite conclusions; larger studies are needed especially in Isfahan that is the concentration zone of MS in Iran. Therefore, tests were planned for more patients.

## Conclusion

–1562 T allele of *MMP-9* is associated with a raised risk of MS susceptibility, so people who carry this polymorphism have an increased risk of MS. However, we found no overall significant effect of alleles with 22 or more CA repeats in the microsatellite polymorphism in the promoter of *MMP9* gene on MS susceptibility. Concerning the significant influence of T allele on MS susceptibility and the non-significant association regarding CA repeats occurred due to the low number of samples evaluated in the present study, further research and experiments are required to determine whether any of *MMP-9* polymorphisms influence MS susceptibility in Iranian MS patients or not. Forasmuch as *MMP-9* molecule is a potential target for MS therapy but the status of *MMP-9* genetic variants on MS susceptibility is disputable, therefore carrying out further research and experiments such as case-control studies with larger samples which analyze effect of different haplotypes on *MMP-9* expression seem necessary before any definite conclusion.

## Ethical considerations

Ethical issues (Including plagiarism, informed consent, misconduct, data fabrication and/or falsification, double publication and/or submission, redundancy, etc.) have been completely observed by the authors.
